# Effect of garlic extract on weight loss and gut microbiota composition in obese women: A double-blind randomized controlled trial

**DOI:** 10.3389/fnut.2022.1007506

**Published:** 2022-10-24

**Authors:** Fateme Ettehad-Marvasti, Hanieh-Sadat Ejtahed, Seyed-Davar Siadat, Ahmad-Reza Soroush, Zahra Hoseini-Tavassol, Shirin Hasani-Ranjbar, Bagher Larijani

**Affiliations:** ^1^Obesity and Eating Habits Research Center, Endocrinology and Metabolism Clinical Sciences Institute, Tehran University of Medical Sciences, Tehran, Iran; ^2^Endocrinology and Metabolism Research Center, Endocrinology and Metabolism Clinical Sciences Institute, Tehran University of Medical Sciences, Tehran, Iran; ^3^Microbiology Research Center, Pasteur Institute of Iran, Tehran, Iran

**Keywords:** gut microbiota, clinical trial, obesity, *Allium sativum*, prebiotic

## Abstract

**Objective:**

From a nutritional perspective, garlic extract could be a prebiotic product, which is useful for obese subjects, and one of its health-promoting underlying mechanisms is modulating gut microbiota composition. In this randomized double-blind clinical trial, the goal was to determine the effect of *Allium* (garlic extract) on anthropometric indices and gut microbiota composition in obese women following a low-calorie diet.

**Materials and methods:**

Forty-three obese women were randomly divided into garlic extract (400 mg *Allium sativum* powder containing 1,100 mcg allicin/tablet) or placebo groups. During the 2 months of the study, each participant took two tablets per day. At the beginning and at the end of the clinical trial, anthropometric measurements were done and blood and fecal samples were collected. We evaluated the gut microbiota composition using quantitative real-time PCR.

**Results:**

In total, 16 subjects in each group completed the 2-month trial. Allium and placebo groups’ participants had mean ages of 37.8 ± 7.4 and 34.2 ± 6.8 years, respectively (*P* > 0.05). Baseline body mass index (BMI) was significantly different between groups, subjects in the placebo group had lower BMI compared with the Allium group (*P* < 0.05). *Allium* and placebo caused a 1.7% and 2.7% decrease in BMI from the baseline values, respectively (*P* < 0.01). Fasting insulin level significantly decreased in the both groups (*P* < 0.01). Level of homeostasis model assessment of insulin resistance (HOMA-IR) has decreased significantly in the Allium group (*P* = 0.007). The frequency of *Akkermansia* had decreasing trend while the abundance of *Faecalibacterium* and *Bifidobacterium* showed increasing trend in the Allium group.

**Conclusion:**

In the both groups, a decrease in BMI and other anthropometric indices has been observed. Despite weight loss after following a low-calorie diet and taking *Allium*, slight changes have been shown in the composition of gut microbiota in obese women.

**Trial registration:**

This trial was registered in the Iranian Registry of Clinical Trials (IRCT) (code: IRCT090420001825N2).

## Introduction

Obesity as a pandemic has challenged the health care systems all over the world. The worldwide prevalence of obesity and overweight has nearly tripled over the past three decades (1, 2). The prevalence of obesity was reported 22% in women within the Middle East and it has been remained stable between 2014 and 2020. 45% of Iranian women suffered from obesity and overweight in 2016 (3–5). Obesity is considered the most common health problem, causing many chronic diseases such as cardiovascular disease, diabetes, inflammation, high blood pressure and cancers (6, 7). Despite the huge investments for the development of effective anti-obesity agents, only a few drugs have been approved for marketing. Furthermore, several of them have been withdrawn due to their side effects. Due to the limited number of safe and effective drugs approved, especially for the long-term treatment of obesity, there is an urgent need to discover new effective weight loss agents.

In recent decades, medicinal plants have become more popular than synthetic drugs and bariatric surgeries due to their higher access and relatively fewer side effects (8, 9). Recent evidence has shown the effects of different species from the genus *Allium* and its bioactive compounds on health, including anti-obesity, anti-diabetic, antioxidant, anti-inflammatory, antimicrobial, antifungal, anti-scar and anti-cancer properties (10–12). Therefore, its prescription along with lifestyle modification may be useful for the prevention and treatment of obesity.

The most studied species of *Allium* whose medicinal properties have been known since ancient times, include *Allium sativum* (garlic), *Allium cepa* (onion), *Allium ampeloprasum var. porrum* (leek) and *Allium ascalonicum* (shallot) (13, 14). Garlic is known as a prebiotic food that is rich in sulfur-containing compounds such as allicin, allian, ajoenes, vinyldithins and flavonoids such as quercetin (15, 16). Allicin (diallyl thiosulfinate) isolated from garlic extract is a lipid-soluble sulfur and natural food compound with numerous biological and medicinal functions. Garlic extract and specifically allicin is known for its weight loss properties, reduction of adipose tissue mass and improvement of plasma lipid profile *via* the downregulation of multiple genes expression that is included in adipogenesis along with upregulation of mitochondrial inner membrane proteins expression (12, 17, 18). Moreover, *Allium* appears to affect the composition of gut microbiota. The gut microbiota as a complex and dynamic microbial community plays an important role as a link between genes, environment and immune system and is associated with diseases such as obesity. Some studies have been evaluated the effect of *Allium* and polyphenolic components on the gut microbiota composition (14, 19–21). However, since the effect of garlic extract on intestinal microbial composition varies in different health situations, it should also be evaluated in healthy obese individuals.

This double-blind randomized controlled clinical trial was conducted in obese women to evaluate the effect of garlic extract alongside a low-calorie diet on anthropometric indices and gut microbiota composition.

## Materials and methods

### Subjects

This randomized, double-blind, parallel-group clinical trial has been conducted at the Obesity Clinic of Shariati Hospital in Tehran, Iran. Advertisements in clinics and hospitals had been done between October 2017 and March 2018 and evaluation according to the eligibility criteria, recruitment of participants and intervention has been started in April 2018. Women between 20 and 45 years old, body mass index (BMI) in the range of 30–40 kg/m^2^, willing to comply with the study criteria, were included in this study. We considered the following criteria for exclusion: pregnancy and lactation, smoking, use of antibiotics, corticosteroids, prebiotics or probiotics during the 3 months before the study and suffering from co-morbidities including cardiovascular disease, kidney and liver disorders, gastrointestinal disorders, inflammatory bowel diseases and cancer and history of acute, chronic diarrhea over the last month. Sample size was determined according to the expected change of 2 kg/m^2^ in BMI between garlic extract and placebo groups. The alpha value and power were considered equal to 0.05 and 80%, respectively. The targeted sample size was calculated as 20 participants per group by considering a 20% drop-out rate.

All participants signed an informed consent form. The ethical committee of Endocrinology and Metabolism Research Institute of Tehran University of Medical Sciences approved all procedures (ID number: IR.TUMS.EMRI.REC.1395.0090) and the trial was registered with the Iranian Registry of Clinical Trials (IRCT) under the code IRCT20090420001825N2.

### Study design

In total, 43 subjects were randomly assigned to the garlic extract (*n* = 21) or placebo (*n* = 22) groups by balanced block randomization using computer random numbers. Patients were given two pills of garlic extract (*Allium*-S, containing 400 mg *Allium* sativum powder equivalent to 1,100 mcg allicin per tablet, Dineh Iran Industrial Complex, Iran) or placebo (containing lactose and starch) per day for 2 months, which they received with the main meals. To reduce the daily caloric intake by 500 kcal, both groups were given a low-calorie diet (LCD) throughout the study. Diet recommendations consisted of 55% carbs, 30% fats, and 15% protein. At first, the needed energy by each person was calculated, after subtracting 500 kilocalories from the daily energy requirement, the number of needed serving size for each food group was provided. Subjects in both groups were advised to partake in physical activity such as a brisk walk for 30 minutes during the day. Instructions on how to take the pills, not changing medications, and avoiding other supplements were given to participants during the initial visit and the study period.

Both subjects and researchers were blinded to the type of treatment they received. *Allium*-S and placebo pills had the same appearance and were separated by a code. All the pills were packed in the identical sequentially numbered, opaque sealed containers to implement the allocation concealment. At the end of the study, participants were asked to bring their remaining pills. Participants were monitored by investigators during weekly phone calls and those who were taking less than 80% of the pills or not following a low-calorie diet were defined as non-compliant and were excluded from the analysis.

### Anthropometric, dietary and biochemical measurements

Every participant completed a demographic questionnaire and dietary intakes, prescribed and non-prescribed medications, and medical history were recorded for all subjects. After collecting demographic information, anthropometric measurements were performed at the beginning and after 2 months. Participants’ weight was measured in light clothing and without shoes with an accuracy of 0.1 kg using a digital scale (Seca, Germany). Height was measured with a precision of 0.5 cm without shoes using a stadiometer. Then BMI has been calculated as body weight in (kg) divided by the square of height (kg/m^2^).

Waist circumference and hip circumference were measured with an accuracy of 0.1 cm. Waist to hip ratio (WHR) was calculated by waist circumference divided by hip measurement, and waist-to-height ratio (WHtR) has been calculated waist circumference divided by height measurement.

At baseline, participants filled out a semi-quantitative food frequency questionnaire (FFQ) consisting of 147 items to assess energy and macronutrient consumption. A dietitian conducted an interview to determine the frequency of food consumption over the past year. An acceptable reliability and validity of the FFQ has been reported (22, 23).

Blood samples were taken from participants after 12–14 h of overnight fasting at the beginning and end of study. The blood serum was immediately separated at room temperature by centrifuging at 1,300 g for 10 min and they were frozen at −80°C until analysis.

Fasting blood sugar (FBS), total cholesterol (TC), triglyceride (TG), high-density lipoprotein cholesterol (HDL), low-density lipoprotein cholesterol (LDL), very low-density lipoprotein cholesterol (VLDL), alanine aminotransferase (ALT), aspartate aminotransferase (AST) and high-sensitivity C-reactive protein (hsCRP) concentrations were measured by Roche kits using an auto-analyzer instrument (Hitachi, Cobas C 311, Roche Diagnostics GmbH). Serum insulin concentration was measured by an enzyme immunoassay kit (Monobind Inc., Lake Forest, CA, USA). Insulin resistance index was calculated by the homeostasis model assessment of insulin resistance (HOMA-IR) equation: HOMA-IR = [FBS (mg/dL) × fasting inulin concentration (mU/L)/405]. Lipoprotein lipase (LPL), glucagon-like peptide 1 (GLP-1) (ZellBio GmbH, Germany) and fasting-induced adipose factor (FIAF) (BioVendor, Germany) were determined by ELISA kits.

Concentrations of fasting blood sugar (FBS), total cholesterol (TC), triglyceride (TG), high-density lipoprotein cholesterol (HDL), low-density lipoprotein cholesterol (LDL), very low-density lipoprotein cholesterol (VLDL), as well as alanine aminotransferase (ALT), aspartate aminotransferase (AST) and high-sensitivity C-reactive protein (hsCRP) were measured by Roche kits using an auto-analyzer instrument (Hitachi, Cobas C 311, Roche Diagnostics GmbH). An enzyme immunoassay kit was used to determine serum insulin concentration (Monobind Inc., Lake Forest, CA, USA). Insulin resistance index was calculated by the homeostasis model assessment of insulin resistance (HOMA-IR) equation: HOMA-IR = [FBS (mg/dL) × fasting insulin concentration (mU/L)/405]. Lipoprotein lipase (LPL), glucagon-like peptide 1 (GLP-1) (ZellBio GmbH, Germany) and fasting-induced adipose factor (FIAF) (BioVendor, Germany) were measured by ELISA kits.

### DNA extraction from fecal samples

Fresh fecal samples have been collected at baseline and 2 months after intervention in sterile cups and brought to the laboratory immediately. Samples were stored at −80°C to determine the fecal microbial quantity, DNA was extracted from 200 mg of each fecal sample, using QIAamp DNA stool mini kit (Qiagen, Hilden, Germany) according to manufacturer’s instructions. The quality and quantity of the extracted DNA were analyzed by NanoDrop ND-8000 (Thermo Scientific, USA), respectively.

### Quantitative real-time PCR analyses

To determine the abundance of bacterial genera including *Prevotella*, *Lactobacillus*, *Bifidobacterium*, *Akkermansia*, *Bacteroides*, *Faecalibacterium*, *Escherichia* and, specific primers were targeted the bacterial 16s rRNA genes (24–30). The sequence of specific primers used in the current study are presented in Table 1.

**TABLE 1:** 16S rRNA gene specific primers for the studied bacterial genera.

Target organism	Forward (5′ to 3′)	Reverse (5′ to 3′)	Amplicon size (bp)	References
***Lactobacillus***	AGCAGTAGGGAATCTTCCA	CACCGCTACACATGGAG	341	(25)
***Bifidobacterium***	TCGCGTCYGGTGTGAAAG	CCACATCCAGCRTCCAC	243	(26)
***Akkermansia***	CAGCACGTGAAGGTGGGGAC	CCTTGCGGTTGGCTTCAGAT	329	(27)
***Bacteroides***	GGTGTCGGCTTAAGTGCCAT	CGGAYGTAAGGGCCGTGC	140	(29)
***Faecalibacterium***	GGAGGAAGAAGGTCTTCGG	AATTCCGCCTACCTCTGCACT	248	(30)
***Escherichia***	CATTGACGTTACCCGCAGAAGAAGC	CTCTACGAGACTCAAGCTTGC	190	(28)
***Prevotella***	CACCAAGGCGACGATCA	GGATAACGCCYGGACCT	283	(24)

The abundance of bacteria fecal samples was analyzed using quantitative real-time PCR based on SYBER green method (LightCycler^®^ 96 SW Roche, Switzerland). Each Quantitative real-time PCR reaction was composed of SYBR Premix Ex Taq II (RR820L; Takara, Japan), each of the specific 16s rRNA primers, and the DNA template. Each qPCR reaction was performed in triplicate using LightCycler^®^ 8-Tube Strips. The amplification program was designed according to the appropriate annealing temperature: an initial denaturation step 1 cycle of 95°C for 60 s, followed by 40 cycles of denaturation at 95°C for 5 s, annealing at 55°C for 30 s, and extension at 72°C for 30 s. Melting curve analysis was carried out after amplification to control the specificity of PCR reaction, followed by 1 cycle at 95°C for 5 s, 60°C for 60 s, and 95°C for 1 s. To calculate the DNA concentration of each bacterium from fecal samples used to standard curves. The standard curve is graphically represented as a semi-log regression line plot of the obtained threshold cycle value vs. log of DNA concentration.

### Measurement of metabolites in fecal samples

Fecal concentrations of short-chain fatty acids (SCFAs) were determined using gas chromatography. At first, about 100 mg of the fecal sample was suspended in 1 ml of saturated NaCl solution (36%). After that, 50 μL of 10.7 μM 2-ethylbutyric acid (Merck, München, Germany) in MQ water was added as internal standard and glass beads were used to homogenize the samples. Next, 150 μl of 96% H_2_SO_4_ was added and SCFAs were extracted in 3 ml of ether. Then, the gathered ether layer was dried by Na_2_SO_4_ (150 mg). 0.5 μl of isolated supernatant was analyzed by gas chromatography-flame ionization detection (GC-FID) method (Agilent). Gas chromatography, with an analytical column of DB FFAP (30 m × 0.53 mm ID, 1.0 μm; Agilent) and helium grade GC (5.6) was used as the carrier gas with a constant flow of 4.2 ml/min. The temperature of the oven was maintained at 100°C for 3 min initially, increased at 4°C per minute to 140°C (isotherm for 5 minutes) and then at 40°C to 235°C (isotherm for 15 min). The ChemStation (Agilent technologies) was used to process the obtained chromatograms. Acetate, propionate and butyrate were measured using calibration curves obtained based on the quantification of the internal standard.

### Statistical analysis

In this study, statistical analysis was performed by IBM SPSS version 26.0 (SPSS Inc., Chicago, IL, USA). P value < 0.05 was considered as the level of significance. Data were expressed as mean ± standard deviation or median (± IQR). The normality distribution for different variables was tested by the Kolmogorov–Smirnov test. Paired samples *t*-test was used to compare the changes within each group. For variables with non-normal distribution, comparison within two groups was done using Wilcoxon Signed Ranks Test. Independent samples *t*-test was employed to assess the differences of variables between the intervention and placebo groups. In case of the normality assumption was violated, Mann–Whitney U test was used.

## Results

### Study participants

**FIGURE 1:**
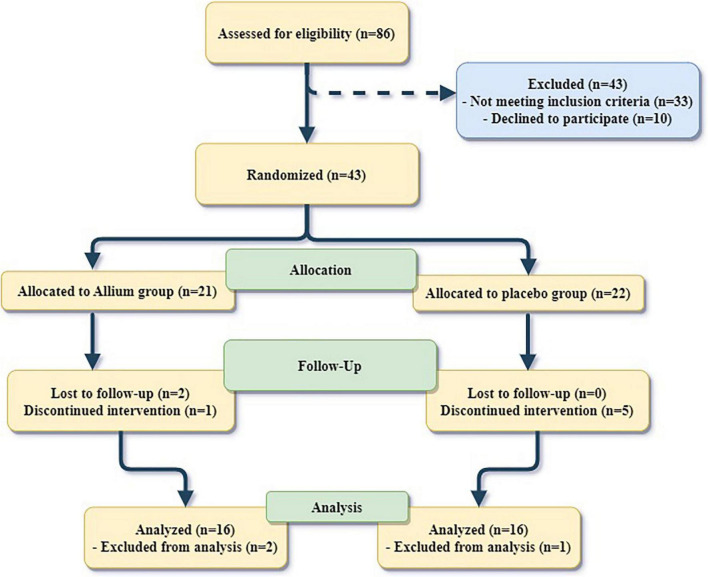
Flow diagram of the study participants.

From the 43 participants, 21 obese women were included in the Allium + LCD group and 22 women were comprised in the Placebo + LCD group. The statistical analysis was conducted on 16 women from each group who finished the 2-month experiment. Two participants were lost to follow-up in the Allium group. Totally, six participants left the trial (one from the Allium group and five from the placebo group) due to a lack of enthusiasm for continuing the interventions and following a low-calorie diet. Moreover, one person in both groups was excluded from analysis because of a lack of procured stool samples (Figure 1). Side effects reported by two participants in the Allium group included itchy skin and heartburn. In the placebo group, two people also complained of bloating and insomnia. There were no differences in the reported side effects between the groups.

The baseline characteristics of the subjects are shown in Table 2. The participant’s mean age in the Allium and placebo groups was 37.8 ± 7.4 and 34.2 ± 6.8 years (*P* > 0.05). BMI was significantly different between groups, subjects in the placebo group had lower BMI compared with the Allium group (*P* < 0.05). No statistically significant difference in body composition, bone mineral density (BMD) and dietary intakes was observed between the study groups at the beginning of the study.

**TABLE 2:** Baseline characteristics and dietary intakes of the study participants.

Variables	Allium (*n* = 16)	Placebo (*n* = 16)
Age (years)	37.8 ± 7.4	34.24 ± 6.82
METS/week	246.0 ± 39	252 ± 38
**Body composition**		
BMI (kg/m^2^)*	35.6 ± 4.4	32.7 ± 2.2
Lean body mass (kg)	46.4 ± 8.8	44.1 ± 3.1
Fat mass (kg)	40.0 ± 10.5	38.1 ± 5.4
Body fat percentage (%)	44.7 ± 3.8	44.9 ± 3.9
BMD (g/cm^2^)	1.18 ± 0.08	1.18 ± 0.07
**Dietary intake**		
Energy (kcal/day)	2768 ± 720	2614 ± 657.5
Carbohydrate (g/day)	420.0 ± 123.0	398.0 ± 118.0
Protein (g/day)	96.0 ± 30.0	94.0 ± 24.0
Fat (g/day)	85.0 ± 25.0	83.0 ± 24.0
Fiber (g/1000 kcal)	19.0 ± 5.0	20.0 ± 7.0

BMI, body mass index; BMD, bone mineral density; WHR, waist-to-hip ratio; WHtR, waist to height ratio; METS, metabolic equivalents.

Data are presented as mean ± SD. Comparisons were made with independent samples *t*-test.

*Significant difference between groups.

### Effect of *Allium* on anthropometric indices in obese women

Changes of the anthropometric indices during the study in obese women are presented in Table 3. Body mass index decreased significantly in both groups from the baseline values. BMI decreased by 1.7% in the Allium group and 2.7% in the placebo group (*P* < 0.01). The BMI reduction was higher in the placebo group than in the Allium group. Weight, waist circumference, hip circumference and WHtR had a significant decrease in both Allium and placebo groups (*P* < 0.01). There were no significant differences between the two groups by adjusting the baseline values (*P* > 0.05).

**TABLE 3:** Effects of 2 months consumption of *Allium* and placebo on anthropometric indices and biochemical variables in obese women.

Variables	Allium (*n* = 16)		Placebo (*n* = 16)		*P*-value
	Baseline	After intervention	Baseline	After intervention	
**Anthropometrics**					
Weight (kg)	91.0 ± 18.25	88.5 ± 21.25*	88.5 ± 9.38	84.5 ± 7.5*	0.21
BMI (kg/m^2^)	35.16 ± 6.96	34.20 ± 9.71*	32.46 ± 3.89	31.04 ± 3.13*	0.46
Waist (cm)	106.0 ± 11.5	98.5 ± 16.0*	100.0 ± 7.75	95.0 ± 7.0*	0.55
Hip (cm)	122.0 ± 14.5	117.0 ± 16.25*	115.0 ± 9.75	110.0 ± 6.5*	0.37
WHR	0.89 ± 0.07	0.87 ± 0.05	0.86 ± 0.09	0.84 ± 0.08	0.54
WHtR	0.66 ± 0.07	0.61 ± 0.09*	0.61 ± 0.03	0.58 ± 0.03*	0.47
**Glucose metabolism**					
FBS (mg/dL)	78.0 ± 13.0	78.0 ± 11.0	79.0 ± 16.5	85.0 ± 9.5	0.99
Insulin (mU/L)	6.7 ± 12.9	4.2 ± 4.6*	7.2 ± 11.2	4.8 ± 4.1*	0.84
HOMA-IR	1.33 ± 2.63	0.95 ± 0.9*	1.33 ± 1.93	1.0 ± 1.2	0.58
**Lipid profile**					
TC (mg/dL)	178.0 ± 36.5	175.0 ± 41.0	182.5 ± 46.25	170.5 ± 33.0	0.08
TG (mg/dL)	134.0 ± 93.0	127.0 ± 116.0	148.0 ± 59.0	149.0 ± 80.25	0.49
LDL (mg/dL)	111.0 ± 45.0	105.0 ± 33.5	114.0 ± 33.5	103.0 ± 29.5	0.06
HDL (mg/dL)	37.0 ± 10.0	41.0 ± 15.5	42.0 ± 11.5	41.5 ± 8.75	0.84
**Liver markers**					
ALT (U/L)	11.0 ± 5.0	12.0 ± 6.0	10.0 ± 2.0	11.5 ± 7.75	0.96
AST (U/L)	19.0 ± 5.0	19.0 ± 4.0	19.0 ± 3.75	18.5 ± 5.75	0.15
**Satiety hormone**					
GLP1 (pg/mL)	29.0 ± 12.0	43.2 ± 284.3	29.1 ± 16.2	86.5 ± 294.53*	0.36
**Inflammatory marker**					
hsCRP (mg/L)	7.2 ± 5.45	5.1 ± 5.2	2.0 ± 2.83	2.4 ± 8.45	0.33
**Adipose factors**					
FIAF (ng/mL)	65.0 ± 37.5	60.0 ± 32.5	70.0 ± 23.5	67.5 ± 13.75	0.84
LPL (pg/mL)	491.7 ± 148.75	297.5 ± 1085.55	426 ± 208.1	494.75 ± 1396.23	0.58

Data are presented as median ± IQR.

ALT, alanine aminotransferase; AST, aspartate aminotransferase; BMI, body mass index; FBS, fasting blood sugar; FIAF, fasting-induced adipose factor; GLP-1, glucagon-like peptide 1; HDL, high density lipoprotein cholesterol; HOMA-IR, homeostasis model assessment of insulin resistance; hsCRP, high sensitive C reactive protein; LDL, low density lipoprotein cholesterol; LPL, lipoprotein lipase; TC, total cholesterol; TG, triglycerides; WHR, waist to hip ratio; WHtR, waist to height ratio.

*Significant difference within groups.

*P*-value indicates significance of comparison between groups.

### Effect of *Allium* on serum concentration of biochemical variables in obese women

Effects of intervention groups on biochemical variables in obese women are shown in Table 3. Fasting blood glucose concentration in the placebo group increased significantly compared to baseline values (*P* = 0.028). Fasting insulin concentration significantly decreased in the both groups after interventions (*P* < 0.01); however, there were no significant differences between the two groups. Level of insulin resistance index (HOMA-IR) had decreased in both groups during the study, although this reduction was significant only in the Allium group (*P* = 0.007). The level of total cholesterol and LDL-C in the placebo group was reduced significantly (*P* < 0.05) and the concentration of LDL-C after the intervention showed a difference between groups (*P* = 0.058). HDL-C value was increased in the Allium group but this increase was not statistically significant (*P* = 0.135). Glp1 concentration had an increasing trend in both groups; however, this increase was only significant in the placebo group (*P* = 0.008). The concentrations of hepatic enzymes, hsCRP, FIAF and LPL factors did not change significantly during the interventions (*P* > 0.05).

### Effect of *Allium* on gut microbiota composition in obese women

**FIGURE 2:**
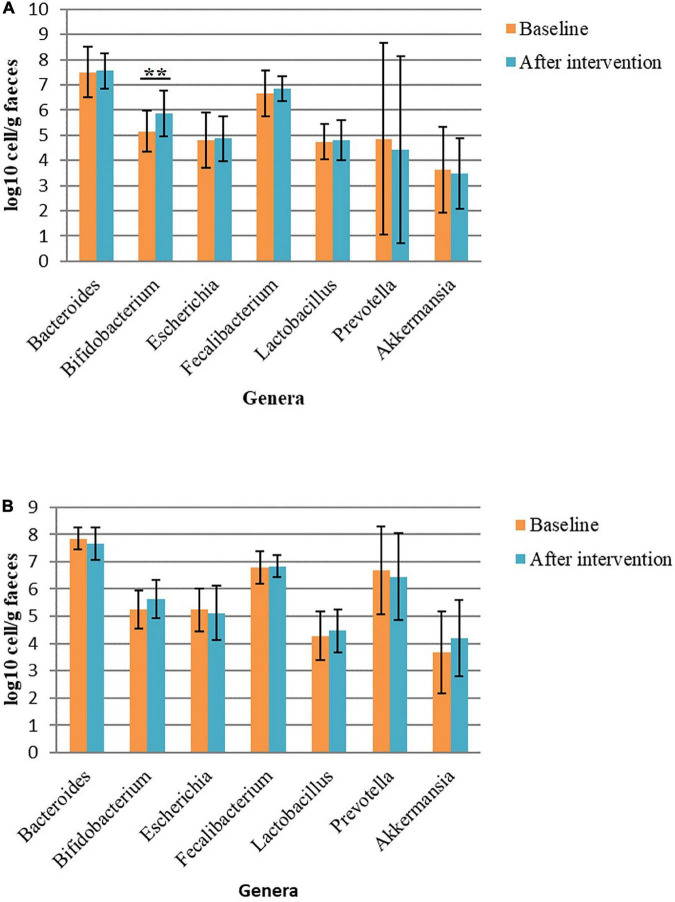
Bacterial load in fecal samples before and after intervention in obese women. **(A)** Bacterial load before and after Allium intake. **(B)** Bacterial load before and after placebo intake. Comparisons were done using paired samples *t*-test or Wilcoxon test. *Significant difference within groups. **p-value < 0.01.

The abundance of some bacterial genera had changed with the intervention. In the Allium group, the abundance of *Bifidobacterium* had increased significantly (*P* = 0.005). Moreover, there were non-significant changes in the frequency of some bacterial genera in this group. The frequency of *Akkermansia* decreased after the intervention and the abundance of *Faecalibacterium*, a butyrate-producing genus, had increased. The frequency of *Akkermansia, Lactobacillus* and *Bifidobacterium* had non-significant increase in the placebo group. Within Bacteroidetes phylum, the bacterial load of *Prevotella* decreased in both intervention groups. No significant difference was found in bacterial level between the two groups (*P* > 0.05) (Figure 2).

### Effect of garlic extract on fecal short-chain fatty acids

**FIGURE 3:**
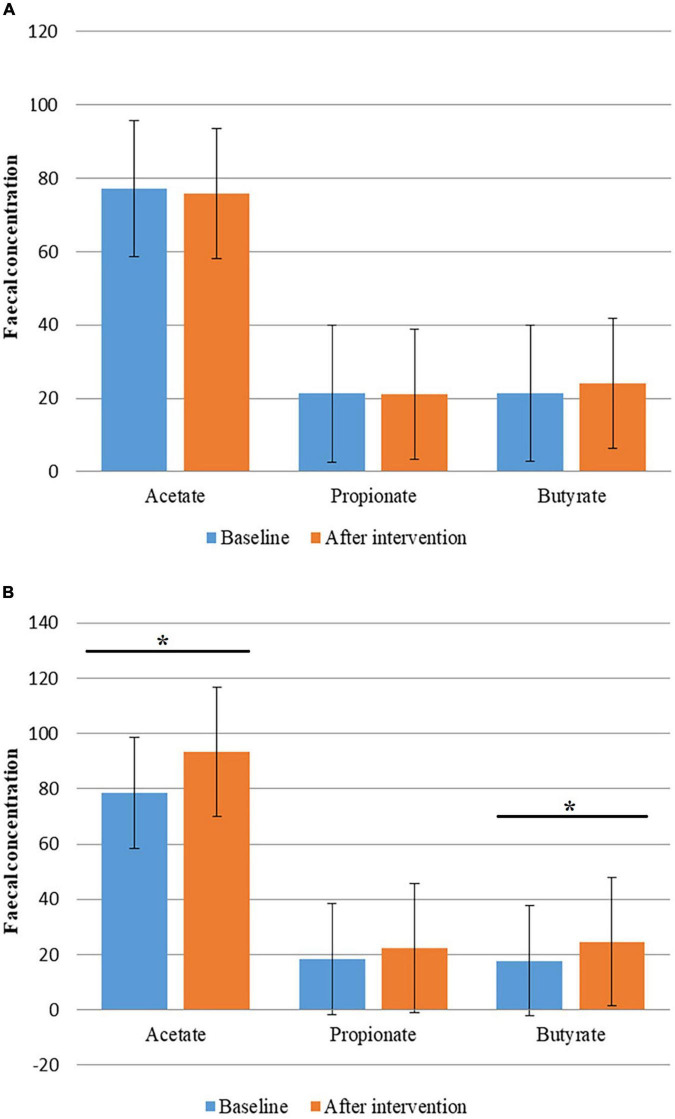
Fecal concentration of short chain fatty acids (SCFAs) before and after intervention in obese women. **(A)** Fecal concentration of SCFAs before and after Allium intake. **(B)** Fecal concentration of SCFAs before and after placebo intake. Comparisons were done using paired samples *t*-test or Wilcoxon test. *Significant difference within groups. *P-value < 0.05.

Although changes in the concentration of fecal SCFAs in the Allium group were not significant; concentration of butyrate had a slight increase. An increase in total SCFAs concentration was observed in the placebo group. Fecal concentrations of acetate and butyrate were significantly increased during the study in the placebo group (*P* < 0.05). Fecal SCFAs levels had no significant difference between the two groups (*P* > 0.05) (Figure 3).

## Discussion

During this single site, randomized, double-blind, placebo-controlled parallel-group clinical trial on obese women, we showed that anthropometric indices and insulin concentration significantly decreased in both garlic extract + LCD and placebo + LCD groups. Besides, a decrease in insulin resistance as well as an increase of HDL-C level have been observed in the Allium group compared to the placebo. We found no significant changes in hepatic enzymes, lipid profile and satiety regulating hormones.

In this study, we observed a significant decrease in BMI, weight, waist and hip circumferences in the Allium group. However, weight reduction was also observed in the placebo group, maybe as a result of low calorie diet. Since *Allium* has been added as a complementary treatment to the low-calorie diet in this study, it seems that a longer intervention period is needed for reaching a clearer conclusion regarding the extra effect of *Allium*. In accordance with our study, Zhang et al. showed that transplantation of Allicin-induced gut microbiota to high fat diet (HFD)-fed mice could prevent body weight and adipose tissue gain and improve plasma lipid profiles and energy homeostasis without any effects on energy intake (31). Aslani et al. indicated that garlic plus lemon juice resulted in an improvement in lipid levels, fibrinogen and blood pressure of patients with hyperlipidemia (32). Joo et al. suggested that high hydrostatic pressure extract of garlic efficiently could reduce body weight gain and lipid profile, partially mediated by regulating lipid metabolism, increasing of the fecal triglyceride excretion and downregulation of adipogenic genes expression and plasminogen activator inhibitor 1 together with upregulation of UCP2, resistin, and TNF-α gene expression in rats fed a high-fat diet (11, 33, 34). Sangouni et al. showed that garlic powder supplementation improved hepatic features and lipid profile among non-alcoholic fatty liver disease (NAFLD) patients (35). However, in our study no significant change was observed in LDL-C and hepatic enzymes. This discrepancy may be due to different doses of used active ingredients and intervention period.

In this study, we observed a significant decrease in insulin concentration and level of insulin resistance in the Allium group. Padiya et al. showed that homogenized raw garlic is effective in improving insulin sensitivity, and significantly reduced serum glucose, insulin, and oxidative stress in fructose-fed rats (36). Sangouni et al. showed that garlic powder supplementation improved insulin resistance and oxidative stress which are the main causes of fatty liver among NAFLD patients (37). Similar to our findings, Maeda et al. observed decrease in insulin resistance in mice which were fed an aged garlic extract supplemented diet. The hypoglycemia effect of garlic is mainly attributed to the presence of active compounds including allicin and dialyl sulfide (38). Considering that weight gain is one of the main factors of insulin resistance (39), we showed that oral administration of garlic extract for two months caused simultaneously a significant decrease in body weight and an improvement of insulin sensitivity. Therefore, reduction of body weight in the present study may contributed in the improvement of insulin sensitivity.

Studies have shown that dietary changes affect the composition of the gut microbiota in animals and humans. In this study, *Allium* supplementation has been associated with an increase in abundance of *Bifidobacterium* and a decrease in abundance of *Bacteroides*; although these changes have not reached statistically significant level, they are clinically important. According to the recent research, the best prebiotic compounds are those which cause an increase of butyrogenic bacteria in addition to increasing the abundance of bifidogenic bacteria. Bifidogenic bacteria produce acetate and lactate, which are consumed by butyrogenic bacteria producing butyrate (40, 41). In the present study, the consumption of *Allium* increased abundance of *Bifidobacterium* as well as *Faecalibacterium*.

The gut microbiota is one of the factors involved in systemic immune response. It has been shown that the frequency of *Bifidobacterium*, *Faecalibacterium*, *Ruminococcus* and *Prevotella* was inversely associated with hsCRP (42–44). In the present study, the increasing trend of *Faecalibacterium* is in accordance with decreasing trend of hsCRP in the Allium group.

Chen et al. (45) showed that whole garlic supplementation increases intestinal microbiome diversity while also decreasing the relative abundance of *Prevotella*. Overall, they showed that garlic supplementation could improve HFD-induced dyslipidemia and intestinal microbiome disorder in the mouse model (45). The results of our study showed a decrease in the frequency of *Prevotella* in line with this study. In another study, Zhang et al. showed that transplantation of Allicin-induced gut microbiota to HFD-fed mice could significantly improve the gut microbiota composition and induced enrichment of *Bifidobacterium* and *Lactobacillus* (31). In our study, we also identified an increasing trend in *Bifidobacterium*, but no significant change was observed in the frequency of *Lactobacillus*. Wu et al. observed that intervention with black garlic melanoidins (MLDs) modified the gut microbiota in HFD-induced obese mice as the frequency of SCFA-producing bacteria (*Bacteroidaceae*) and probiotics bacteria including *Lactobacillaceae* and *Akkermansiaceae* was increased and the frequency of opportunistic pathogens (*Enterobacteriaceae* and *Desulfovibrionaceae*) was decreased. Therefore, MLDs can improve glucose tolerance, induce SCFAs production and inhibit the production of LPS endotoxin, most likely by modulating the gut microbiota (46). In contrast, the results of our study showed a slight decrease in the frequency of *Akkermansia* in the Allium group which may have occurred as a result of changes in food intake. More and longer studies are needed in this field to differentiate the effects of diet and *Allium*.

Recent research has shown that H_2_S gas, which is produced by the activity of sulfate-reducing bacteria in the colon, can directly stimulate the production of GLP-1 and could be effective in controlling appetite. This gas is produced from fermentation of organosulfur prebiotics (47, 48). In this study, we also saw an increase in the concentration of GLP-1 as a consequence of sulfur-containing Alliin intake. The effect of this compound on sulfate-reducing bacteria in the gut needs to be further investigated. About 80% of garlic organosulfur compounds are alliin, which are transformed to allicin.

Previous studies have proposed increased production of SCFAs *via* fermentation of intestinal bacteria as a potential mediating mechanism of health properties of garlic extract (46, 49). However, in our study the concentration of SCFAs did not change significantly. Yuan et al. showed that allicin regulated cascade response of the microbiota-SCFAs signaling to reverse the reduction of acetic acid and propionic acid by acryl amid treatment (50). Sánchez et al. showed that *Allium* extract increased the levels of propionic, isobutyric, and isovaleric acids significantly (51).

In the placebo group, anthropometric indices had a decreasing trend and energy restriction in this group improved blood insulin, total cholesterol and LDL and increased GLP1. The diet prescribed in this study was mild and only reduced about 500 kcal of the participants’ energy intake, therefore may have a limited effect on the gut microbiota composition in the placebo group. The results of microbiota assessment in this group showed that the frequency of *Akkermansia* and *Bifidobacterium* had an increasing trend but did not reach a significant level. The increasing trend of *Bifidobacterium* is in line with increased fecal butyrate concentration in this group. Evidence has shown the anti-inflammatory properties of butyrate which has a beneficial effect on gut health (52). To confirm the observed results, further studies with higher sample sizes and longer intervention periods are needed. In order to measure the extent to which the dietary intervention makes the composition of the microbiota of obese women similar to that of healthy people with normal weight, considering a healthy group in future studies could be helpful. Moreover, it should be noted that prescribed diet was the same in both groups, so some specific changes in composition of microbiota could be expected as a result of *Allium* addition.

To the best of our knowledge, this is the first clinical trial to examine the effect of *Allium* on the composition of the gut microbiota of obese women. One of the strengths of this study is its design as a double-blind randomized clinical trial. Considering that the composition of the gut microbiota of each individual is compared before and after the intervention, the effects of interpersonal differences of microbiota are eliminated. Moreover, the effect of two important confounders including antibiotic and slimming drugs has been eliminated by considering as exclusion criteria. This study has some limitations. We cannot determine how much of the observed weight loss is linked to microbiota changes. As an additional limitation, this study did not include a group of healthy controls, so we did not compare the gut microbiota changes due to *Allium* with microbiota composition of healthy individuals. Another weakness of this study is its low sample size. Further clinical trials with higher sample size and greater power are recommended in this regard. In the present study, *Allium* has been prescribed along with the LCD, so a longer intervention period may have been required in order to distinguish the diet and *Allium* effects.

## Conclusion

In the present clinical trial, *Allium* supplementation along with low calorie diet caused weight loss in obese women during 2 months of intervention and the frequency of fecal *Bifidobacterium* and *Faecalibacterium* in the Allium group had increasing trend. Further studies are needed to investigate the correlation between weight changes and gut microbiota alterations as a result of garlic extract supplementation.

## Data availability statement

The data that support the findings of this study are included in the article/Supplementary material.

## Ethics statement

The studies involving human participants were reviewed and approved by the Ethical Committee of Endocrinology and Metabolism Research Institute of Tehran University of Medical Sciences (ID number: IR.TUMS.EMRI. REC.1395.0090). The patients/participants provided their written informed consent to participate in this study. Written informed consent was obtained from the individual(s) for the publication of any potentially identifiable images or data included in this article.

## Author contributions

H-SE and BL proposed the concept of the study. BL, S-DS, and SH-R designed the study. ZH-T, FE-M, A-RS, and H-SE did the data collection or analysis. H-SE and S-DS performed the interpretation. FE-M and H-SE wrote the study. All authors have read and approved the manuscript.
